# Molecular detection of tick-borne pathogens in caracals (*Caracal caracal*) living in human-modified landscapes of South Africa

**DOI:** 10.1186/s13071-020-04075-5

**Published:** 2020-04-30

**Authors:** Storme Viljoen, M. Justin O’Riain, Barend L. Penzhorn, Marine Drouilly, Laurel E. K. Serieys, Bogdan Cristescu, Kristine J. Teichman, Jacqueline M. Bishop

**Affiliations:** 1 Institute for Communities and Wildlife in Africa, Department of Biological Sciences, University of Cape Town, Cape Town, South Africa; 2 Vectors & Vector-borne Diseases Research Programme, Department of Veterinary Tropical Diseases, University of Pretoria, Pretoria, South Africa; 3 National Zoological Garden, South African National Biodiversity Institute, Pretoria, South Africa; 4 The Cape Leopard Trust, Cape Town, South Africa; 5 Department of Biology, University of British Columbia, Kelowna, Canada

**Keywords:** *Anaplasma*, *Babesia felis*, *Babesia leo*, *Hepatozoon felis*, Reverse Line Blot Hybridisation

## Abstract

**Background:**

Wild carnivores living alongside humans and domestic animals are vulnerable to changes in the infectious disease dynamics in their populations. The aims of this study were to determine the prevalence and diversity of selected tick-borne pathogens (TBPs) of veterinary and/or zoonotic concern in wild populations of caracals (*Caracal caracal*) occurring in human-modified landscapes in South Africa. Using molecular techniques, we screened 57 caracal blood samples for infection by rickettsial bacteria and piroplasms in three regions of South Africa: rangeland in the Central Karoo (*n* = 27) and Namaqualand (*n* = 14) as well as the urban edge of the Cape Peninsula (*n* = 16) of South Africa. To characterise pathogen identity, we sequenced the *18S* rRNA and *16S* rRNA genes from positive samples and analysed sequences within a phylogenetic framework. We also examine the diversity of potential tick vectors.

**Results:**

All individuals tested were infected with at least one tick-borne pathogen. Pathogens included *Hepatozoon felis*, *Babesia felis*, *Babesia leo* and a potentially novel *Babesia* species. An *Anaplasma* species previously described in South African domestic dogs was also found in 88% of urban edge caracals. Higher rates of co-infection characterised urban edge caracals (81% *vs* 15% and 0% in the two rangeland populations), as well as a greater incidence of mixed infections. Host attached tick species include *Haemaphysalis elliptica*, an important pathogen vector among carnivore hosts.

**Conclusions:**

This study confirms the occurrence of previously undocumented tick-borne pathogens infecting free-ranging caracals in human-modified landscapes. We identify clear differences in the pathogen profiles among our study populations and discuss the likely health costs to caracals living adjacent to urban areas.
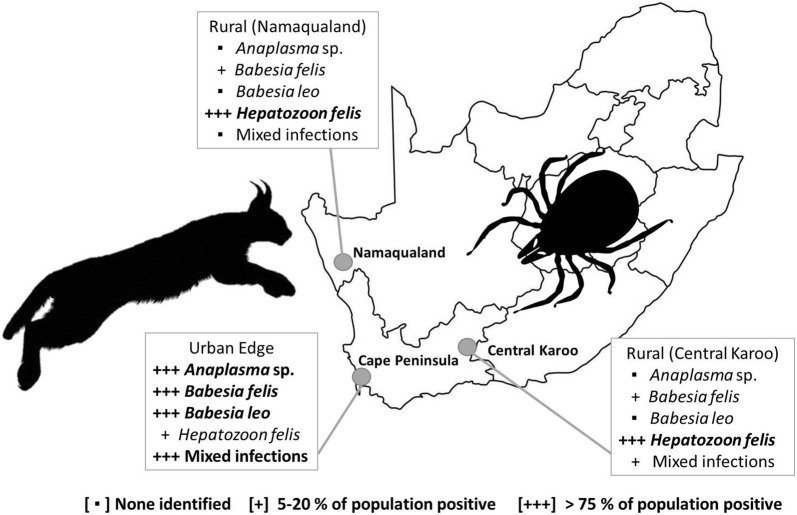

## Background

Landscape modification, and urbanisation specifically, is considered a key driver of emerging infectious diseases in wildlife [1]. Evidence for the negative impacts of urbanisation on wildlife health is emerging [1–5] and across a range of human-modified landscapes, a growing body of literature suggests that disease prevalence can vary substantially in response to environmental change [6]. Detailed investigation into how landscape modification influences both pathogen prevalence and wildlife health is clearly necessary for understanding the complex disease ecologies associated with human-wildlife interfaces.

Much of what is currently understood about the dynamic between landscape modification and wildlife ecology comes from the study of wild carnivores [7–10]. For small to medium-sized carnivores, this is likely due to their ability to persist in landscapes that have undergone varying degrees of anthropogenic change [11, 12]. The caracal (*Caracal caracal*) is an adaptive generalist that is prevalent in both rural [13] and urban [4] environments although its abundance across most of its range is thought to be low [14]. To date there have been no extensive studies on the health of caracals in human-modified landscapes but opportunistic sampling of individuals in disease studies of other felid species have yielded novel findings, specifically for tick-borne pathogens (TBPs) [15, 16].

Despite studies indicating that TBP infections may be common in wildlife species [17, 18], the literature tends to focus on domestic animals and ranched wildlife as TBPs are among the most important disease-causing agents in humans and domestic animals [19, 20]. TBPs vary in their presentation from sub-clinical to acute infection with the vertebrate host presenting fever, lethargy, malaise, jaundice and anorexia [21–25]. Severe infection is also associated with stress factors such as age, immunological status and concomitant infections [24, 26]. In rare instances, infection can be fatal, e.g. hepatozoonosis reported in spotted hyenas (*Crocuta crocuta*) in the Serengeti region of Tanzania [27]. However, very few studies on TBP prevalence in wildlife species have noted clinical manifestations of disease (with the exception of significant wildlife population declines through interactions with other pathogens, e.g. [21, 22, 28]) and are instead concerned about the potential for wildlife to act as reservoir hosts to sympatric domestic animals [29, 30].

Commonly considered in studies of TBP infection in mammalian hosts are pathogens in the orders Rickettsiales (e.g. *Anaplasma* and *Ehrlichia* species), Eucoccidia (e.g. *Hepatozoon* species) and Piroplasmida (e.g. *Babesia*, *Theileria* and *Cytauxzoon* species). Pathogens in the family Anaplasmataceae, which include *Anaplasma* and *Ehrlichia* species, are globally recognised as causative agents of numerous emerging infectious diseases [31–33] and have been reported in wild carnivores, including *Leopardus tigrinus* and *Speothus venaticus* [18]. However, few studies have been carried out specifically to survey these species by molecular means, e.g. [10, 32–36]. In sub-Saharan Africa, wildlife infections by rickettsial bacteria are gaining attention as wildlife managers focus on disease dynamics in reservoir or spill-over populations, e.g. African buffaloes (*Syncerus caffer* [37] and lions (*Panthera leo* [38]).

Infection by the protozoan pathogens, *Hepatozoon*, *Babesia*, *Theileria* and *Cytauxzoon* is relatively well-researched in wildlife [22, 24, 39–41]. In South Africa, *Babesia* infections in wildlife populations have been confirmed in numerous carnivore species, e.g. African wild dogs (*Lycaon pictus* [42]); meerkats (*Suricata suricatta* [43]), and particularly in wild felids including cheetahs (*Acinonyx jubatus*), lions [16], leopards (*Panthera pardus*), black-footed cats (*Felis nigripes*) and caracals [15, 16, 44, 45]. *Hepatozoon* spp. appear to have a worldwide distribution in carnivores, and are reported in numerous species of wild canids and felids in the areas that have been surveyed [25, 34, 46–49]. Less common are reports of *Cytauxzoon* species in African felids [38], although this is a regionally dominant TBP in North America [50] and parts of Europe [41].

Here, we explore how TBP profiles vary with landscape modification by humans in a free-ranging mesocarnivore, the caracal. Using a molecular diagnostic approach, we characterise TBP prevalence and diversity in three caracal populations with the goal of assessing whether pathogen prevalence and co-infection differ between host populations inhabiting two land-use types. We also present our findings of tick species presence found on sampled caracals. To our knowledge, this study represents the first detailed examination of tick-borne pathogens in caracals, a little-studied predator of economic and ecological significance in the southern African region.

## Methods

### Study sites and sample collection

Whole blood samples were collected from caracals in three regions of South Africa: the Central Karoo (−32.66, 22.25), Namaqualand (−30.12, 17.84) and the Cape Peninsula (−34.08, 18.40). The first two sites (“rangeland”) are semi-arid and rural with low human densities and the dominant land-use being free-range small-stock farming. The Namaqualand site includes a section of Namaqua National Park. The Cape Peninsula (“urban edge”) is characterised by a national park (Table Mountain National Park) surrounded by the large metropole of the City of Cape Town and has much higher mean annual precipitation (MAP) than the rangeland sites. The climate on the Cape Peninsula is classified as Mediterranean with hot, dry summers and cold, wet winters. Rainfall in this region is moderate, with a MAP of 480–540 mm. Average minimum and maximum temperatures are 7–18 °C in winter and 16–26 °C in the summer months. The Central Karoo falls within the Nama Karoo biome and is characterised by a continental climate, with highly variable annual rainfall patterns [51]. Most of the rain falls in the late summer, and MAP ranges from 50–240 mm with a maximum of 750 mm on the top of mountain ranges [51, 52]. Average temperatures range between −5–17 °C in winter and 15–43 °C in summer. Drought in this region is common and may persist for prolonged periods [53]. Namaqualand falls within the Succulent Karoo Biome, which has a Mediterranean climate, characterised by winter rainfall [54]. Most rainfall occurs between May and September, with the peak in June [55]. MAP is approximately 160 mm, although some years receive less than 100 mm. Temperatures range between 7–17 °C in winter and 15–30 °C in summer.

Blood sampling in the Central Karoo region took place in April 2015, while sampling in Namaqualand and the Cape Peninsula took place during 2014/2015, spanning 12–16 months across all seasons. Samples from the Central Karoo (*n* = 27) were obtained from individuals culled during permitted annual predator control operations on privately owned farms. Necropsies were performed within 24 h of death. Blood samples were taken from the right ventricle of the heart and stored in EDTA at −20 °C. Samples from Namaqualand (*n* = 14) and the Cape Peninsula (*n* = 16) were collected from live animals under anaesthetic with veterinary assistance in addition to three blood samples collected from vehicle mortalities on the Cape Peninsula (see [4] for details of blood collection for the Cape Peninsula caracals).

Where evident, approximately two to five ticks were removed from each caracal and stored in 70–96% ethanol. Identification to species level was done using a Leica EZ4D stereo-microscope (Leica Microsystems). Species identification was based on field guides [56, 57] and confirmed by Professor Ivan Horak, a recognised expert in African tick identification.

### Pathogen screening by reverse line blot (RLB) hybridisation

All blood samples were first screened for infection by a range of tick-borne pathogens using reverse line blot (RLB) hybridisation. All samples that were positive for species-specific pathogens were then screened using pathogen-specific PCR primers and direct sequencing. Total DNA was extracted from whole blood using the QIAamp® DNA Mini kit (Qiagen, Hilden, Germany) following the manufacturer’s instructions. Multiplex PCR-based RLB hybridisation [58] was used to diagnose infection with a range of tick-borne pathogen species (Table 1). To avoid cross-reaction of primers during PCR, initial amplification was performed separately for *Babesia*/*Theileria* species and *Ehrlichia*/*Anaplasma* species. Although *Babesia*/*Theileria* probes were designed for these genera, they are also able to detect *Cytauxzoon* and *Hepatozoon* species [38, 59]. The *Babesia*/*Theileria* PCR was carried out using primers RLB-F2 (5ʹ-GAC ACA GGG AGG TAG TGA CAA G-3ʹ) and RLB-R2 (biotin-5ʹ-CTA AGA ATT TCA CCT CTG ACA GT-3ʹ) which amplify the V4 region in the *18S* rRNA gene [60, 61]. For the *Ehrlichia*/*Anaplasma* PCR, primers Ehr-F (5ʹ-GGA ATT CAG AGT TGG ATC MTG GYT CAG-3ʹ) and Ehr-R (5ʹ-Biotin-CGG GAT CCC GAG TTT GCC GGGACT TYT TCT-3ʹ) were used to amplify the V1 hypervariable region of the *16S* rRNA gene [62, 63]. *Babesia bovis* and *Anaplasma centrale* from commercial vaccines (Onderstepoort Biological Products, Pretoria, South Africa) were used as positive controls. PCRs were performed in a final reaction mixture of 25 μl, with 12.5 μl of Quantitative PCR Supermix-UDG (Thermo Fisher Scientific, Johannesburg, South Africa); 1 μM stock of each forward and reverse primer; nuclease-free water and up to 5 μl of DNA template. PCR conditions followed a touchdown protocol consisting of an initial step of 3 min at 37 °C, followed by a 10-min step at 94 °C, two cycles at 94 °C (20 s), 67 °C (30 s) and 72 °C (30 s), which were repeated with the annealing temperature decreased by 2 °C until this reached 59 °C. Thereafter, 40 cycles of 94 °C (20 s), 57 °C (30 s) and 72 °C (30 s) were carried out before a final extension step at 72 °C for 7 min.

**Table 1:** Oligonucleotide probes used to detect tick-borne pathogen species in caracals (*Caracal caracal*)

Species	Sequence (5ʹ–3ʹ)	Reference
Order Rickettsiales		
*Ehrlichia/Anaplasma* spp. catch all	GGG GGA AAG ATT TAT CGC TA	[63]
*Ehrlichia canis*	TCT GGC TAT AGG AAA TTG TTA	[106]
*Ehrlichia ruminantium*	AGT ATC TGT TAG TGG CAG	[63]
*Anaplasma bovis*	GTA GCT TGC TAT GRG AAC A	[62]
*Anaplasma centrale*	TCG AAC GGA CCA TAC GC	[63]
*Anaplasma chaffeensis*	ACC TTT TGG TTA TAA ATA ATT GTT	[63]
*Anaplasma marginale*	GAC CGT ATA CGC AGC TTG	[63]
*Anaplasma phagocytophilum*	TTG CTA TAA AGA ATA ATT AGT GG	[62]
*Anaplasma* sp. *Omatjenne*	CGG ATT TTT ATC ATA GCT TGC GCT	[63]
Order Piroplasmida		
*Theileria/Babesia* spp. catch all	ATT AGA GTG TTT CAA GCA GAC	Nijhof (unpublished)
*Theileria* spp. catch all	ATT AGA GTG CTC AAA GCA GGC	[107]
*Babesia* spp. catch all 1	ATT AGA GTG CTC AAA GCA GGC	Nijhof (unpublished)
*Babesia* spp. catch all 2	ACT AGA GTG TTT CAA ACA GGC	Nijhof (unpublished)
*Theileria annae (= Babesia vulpes)*	CCG AAC GTA ATT TTA TTG ATT G	[108]
*Theileria annulata*	CCT CTG GGG TCT GTG CA	[60]
*Theileria bicornis*	GCG TTG TGG CTT TTT TCT G	[109]
*Theileria buffeli*	GGC TTA TTT CGG WTT GAT TTT	[110]
*Theileria equi*	TTC GTT GAC TGC GYT TGG	[111]
*Theileria lestoquardi*	CTT GTG TCC CTC CGG G	[112]
*Theileria mutans*	CTT GCG TCT CCG AAT GTT	[60]
*Theileria ovis*	TGC GCG CGG CCT TTG CGT T	[62]
*Theileria parva*	GGA CGG AGT TCG CTT TG	[60]
*Theileria separata*	GGT CGT GGT TTT CCT CGT	[112]
*Theileria* sp. *buffalo*	CAG ACG GAG TTT ACT TTG T	[113]
*Theileria* sp. *kudu*	CTC CAT TGT TTC TTT CCT TTG	[114]
*Theileria* sp. *sable*	GCT GCA TTG CCT TTT CTC C	[114]
*Theileria taurotragi*	TCT TGG CAC GTG GCT TTT	[60]
*Theileria velifera*	CCT ATT CTC CTT TAC GAG T	[60]
*Babesia bicornis*	TTG GTA AAT CGC CTT GGT	[109]
*Babesia bigemina*	CGT TTT TTC CCT TTT GTT GG	[60]
*Babesia bovis*	CAG GTT TCG CCT GTA TAA TTG AG	[60]
*Babesia caballi*	GTT GCG TTK TTC TTG CTT TT	[115]
*Babesia canis*	TGC GTT GAC GGT TTG AC	[115]
*Babesia divergens*	ACT RAT ATC GAG ATT GCA C	[115]
*Babesia felis*	TTA TGC GTT TTC CGA CTG GC	[16]
*Babesia gibsoni*	TAC TTG CCT TGT CTG GTT T	[108]
*Babesia lengau*	CTC CTG ATA GCA TTC	[45]
*Babesia leo*	TTA TGC TTT TCC GAC TGG C	[16]
*Babesia microti*	GRC TTG GCA TWC TCT GGA	[115]
*Babesia occultans*	CCT CTT TTG GCC CAT CTC GTC	[116]
*Babesia rossi*	CGG TTT GTT GCC TTT GTG	[115]
*Babesia *sp. *sable*	GCG TTG ACT TTG TGT CTT TAG C	[64]
*Babesia vogeli*	AGC GTG TTC GAG TTT GCC	[61]

RLB hybridisation was then performed on PCR product diluted in 2× SSPE/0.1% SDS buffer, following the protocol described by [60], with modifications from [61]. In place of ECL hyperfilm, X-ray film was used and exposed to the chemi-luminescent membrane for 1–3 s. The list of oligonucleotide probes used is detailed in Table 1.

### Diagnostic PCR and cloning for direct sequencing of isolates

A subset of positive samples (minimum 25% per group based on RLB results, e.g. *Anaplasma* isolates) was then selected for PCR and direct sequencing. Where there were fewer than ten positive samples in a species category, all samples were processed. Samples were selected according to the quantity of pathogen DNA that could be obtained. Samples reacting to the generic *Babesia/Theileria* catch-all probes in the RLB assay were amplified using primers Nbab 1F and TB Rev [63, 64] targeting a 1600 bp region of the *18S* rRNA gene. Reactions were performed in a final volume of 25 μl, consisting of using DreamTaq Green PCR Master Mix (2×) (Thermo Fisher Scientific, Johannesburg, South Africa) and 0.5 μM of each primer. Cycling conditions were as follows: 2 min at 95 °C, 30 s each at 95 °C, 31 °C, 72 °C (35 cycles), and a final extension step of 10 min at 72 °C. An additional set of *Babesia/Theileria/Hepatozoon* primers (BTF1 and BTR2) was used as a diagnostic tool for comparison with the RLB results on all samples. The BTF1 and BTR2 primers target a ~ 848 bp fragment of the *18S* rRNA gene [38, 65, 66] and are able to detect *Babesia*, *Theileria*, *Cytauxzoon* and *Hepatozoon* species [38]. Reactions were carried out in a final volume of 25 μl using DreamTaq Green PCR Master Mix (2×) (Thermo Fisher Scientific) and 2.5 μM each of each primer [67].

Samples positive for *Ehrlichia* and *Anaplasma* species were amplified for sequencing using the primers fD1 and rP2 that target a ~ 1600-bp fragment of the *16S* rRNA gene [68]. Reactions were performed in a final volume of 25 μl using DreamTaq Green PCR Master Mix (2×) (Thermo Fisher Scientific) and 1 μM of each primer. All PCR reactions were carried out on an ABI2720 Thermal Cycler (Applied Biosystems, Johannesburg, South Africa) and amplified products were visualised by ethidium bromide staining on 1% agarose gel by electrophoresis. After separation, bands were excised and cleaned using the Wizard SV Gel and PCR Clean-up System (Promega, Madison, USA).

Where direct sequencing suggested infection with multiple pathogen species, samples were cloned using the pGEM-T Easy Vector System I (Promega) and JM109 competent cells (Promega). Blue-white screening was used to detect positive clones and each reaction was plated in duplicate. A minimum of five positive colonies, along with a negative colony, were selected for colony PCR amplification using commercially available plasmid-specific primers, M13-F (5ʹ-GTA AAA CGA CGG CCA GT-3ʹ) and M13-R (5ʹ-CAG GAA ACA GCT ATG AC-3ʹ) (Thermo Fisher Scientific). PCR was performed in a final volume of 20 μl using DreamTaq Green PCR Master Mix (2×) (Thermo Fisher Scientific) and 1 μM each of primers M13-F and M13-R. PCR was performed with the following conditions: 2 min at 95 °C, 35 cycles of 30 s at 95 °C, 20 s at 50 °C, 40 s at 72 °C and a final step for 10 min at 72 °C. Amplified PCR products were gel purified, using a Wizard SV gel and PCR clean-up system (Promega) and sequenced using the M13-F primer.

All DNA sequencing was performed using the Big Dye Terminator Cycle Sequencing Kit version 3.1 on an ABI3730xl Genetic Analyser (Applied Biosystems). Sequencing was performed at the Central Analytical Facility, University of Stellenbosch, South Africa. Sequence chromatograms were visualised and manually edited in BioEdit Sequence Alignment Editor v. 7.2.5 [69].

### Phylogenetic analysis to determine pathogen identity

DNA sequences were analysed using both the Basic Local Alignment Search Tool (BLASTn) function in GenBank and phylogenetic methods to determine their identity in relation to known species. Sequence identity was first assessed using BLASTn, with sequence identity verified at a threshold of 97% [70]. Reference sequences of known identity were selected from GenBank and analysed in conjunction with sequences from this study using a phylogenetic framework. Sequences were first aligned using ClustalW [71] implemented within BioEdit v.7.2.5. Final alignments were trimmed to the length of the shortest sequence and analysed in MEGA v.6.0. I [72]. Phylogenetic trees were generated using both the neighbour-joining and maximum likelihood methods of tree construction. Model selection was performed in MEGA v.6.0.1 and the model with the lowest AICc value was selected [73]. Node support was evaluated with 1000 bootstrap replicates. Outgroups were selected following existing literature [24, 74, 75].

Pathogen prevalence was calculated as the proportion of the total population sample with a positive result for a pathogen. Clopper-Pearson confidence intervals (CI) for binomial response data were calculated using the ‘exactci’ function in the *PropCIs* package in R [76]. Chi-square tests of homogeneity were used to test for differences in prevalence among groups. For multiple comparisons, *P*-values were subjected to Benjamini-Hochberg correction using the *fifer* package in R [77]. All statistical analyses were carried out in R for Windows v 3.2.2 [78].

## Results

A total of 57 caracals from three sites were surveyed for tick-borne pathogens (16 in Cape Peninsula; 27 in Central Karoo and 14 in Namaqualand). Based on the RLB hybridisation all individuals, from all three sites, showed evidence of infection with at least one TBP (see Table 2 for list of pathogens examined).

**Table 2:** Reverse line blot hybridisation detection of tick-borne pathogens in South African caracals (*Caracal caracal*)

Order		Central Karoo (*n* = 27)	Namaqualand (*n* = 14)	Cape Peninsula (*n* = 16)
	Total proportion of positive reactions^a^	100	100	100
	Pathogen: Catch-all probes			
Rickettsiales	*Ehrlichia/Anaplasma* spp.	11 (2–29%)^b^	0 (0–23%)	88 (62–98%)
Piroplasmida	*Theileria/Babesia* spp.	4 (1–19%)	43 (18–71%)	88 (62–98%)
	*Theileria* spp.	0 (0–13%)	0 (0–23%)	31 (11–59%)
	*Babesia* sp. 1	100 (87–100%)	100 (77–100%)	100 (79–100%)
	*Babesia* sp. 2	93 (76–99%)	100 (77–100%)	100 (79–100%)
	Pathogen: Species-specific probes			
	*Babesia felis*	0 (0–13%)	0 (0–23%)	75 (48–93%)
	*Babesia microti*	0 (0–13%)	0 (0–23%)	88 (62–98%)
	*Babesia leo*	0 (0–13%)	0 (0–23%)	63 (35–85%)
	*Theileria annulata*	4 (1–19%)	0 (0–23%)	0 (0–21%)

^a^Pathogens for which there were no positive results are not shown in this table. For an exhaustive list of pathogens, see Table 1

^b^Confidence intervals (95% CI) are calculated according to the Clopper-Pearson method

### Phylogenetic identity of tick-borne pathogens in South African caracals

#### Rickettsial infections

**Fig. 1:**
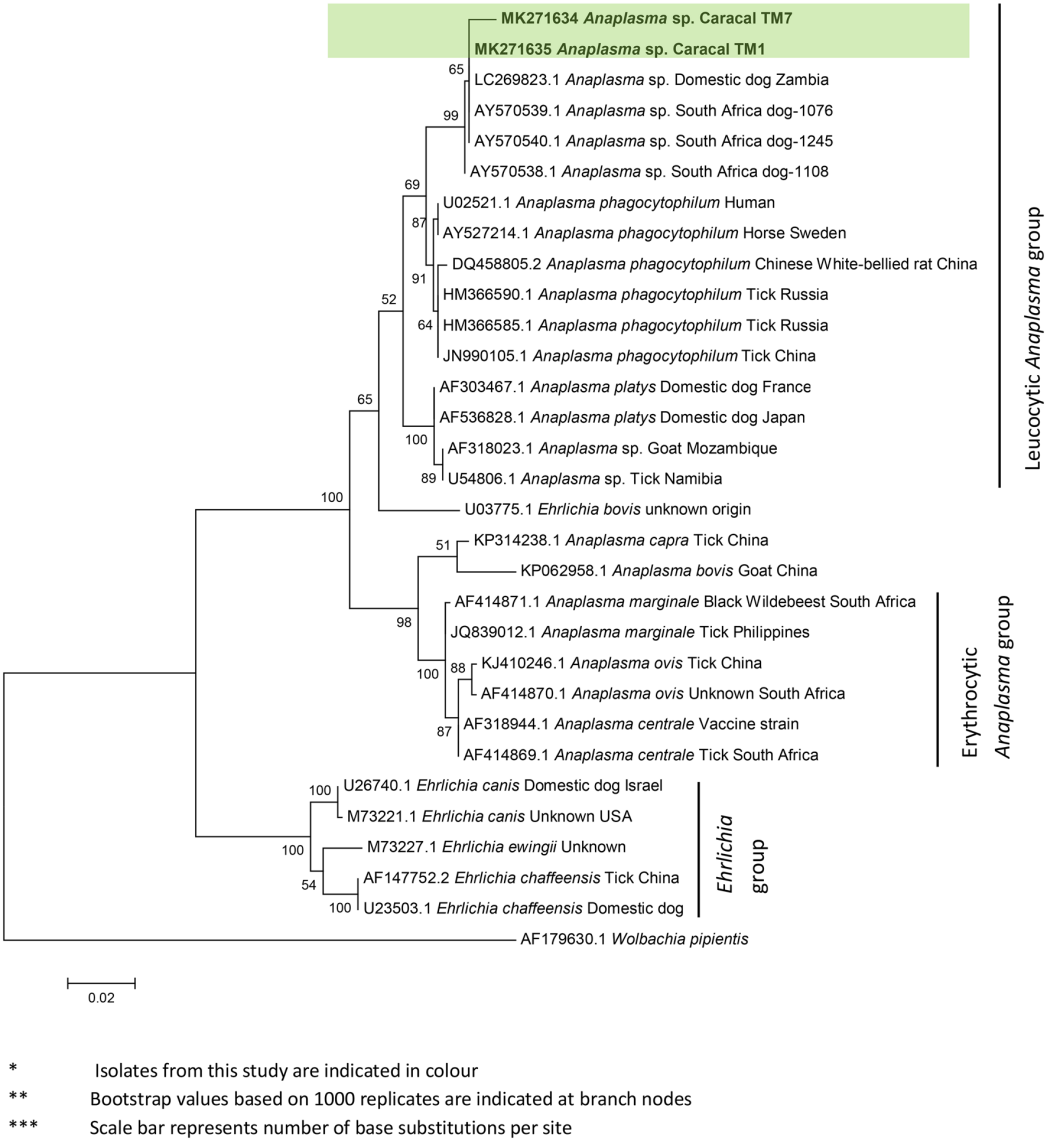
Maximum likelihood phylogeny (K2 + G substitution model) of partial *Ehrlichia* and *Anaplasma* sp. *16S* rRNA gene sequences (849 bp)

**Fig. 2:**
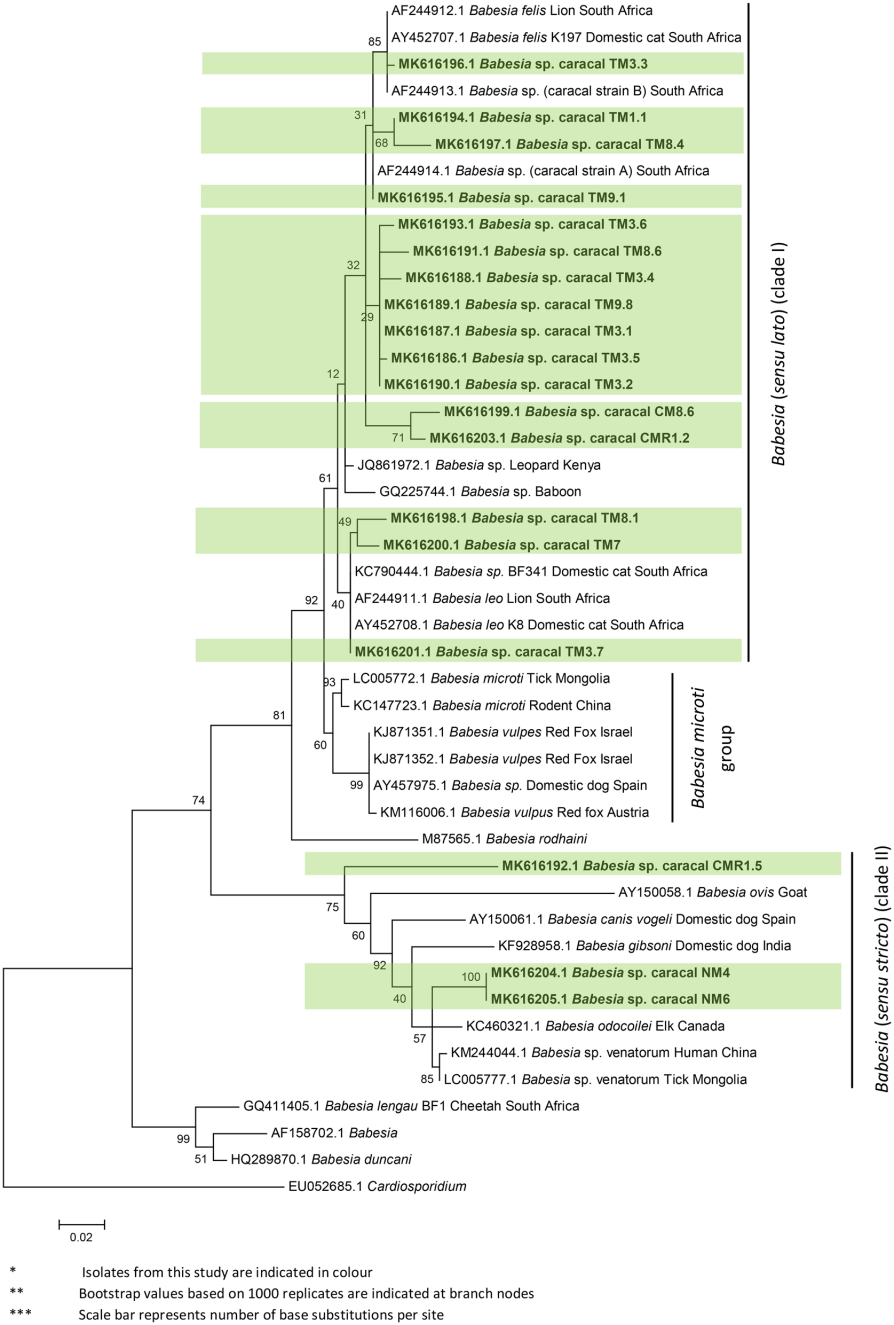
Maximum likelihood phylogeny (T92 + G + I) of partial *Babesia* spp. *18S* rRNA gene sequences (489 bp)

**Fig. 3:**
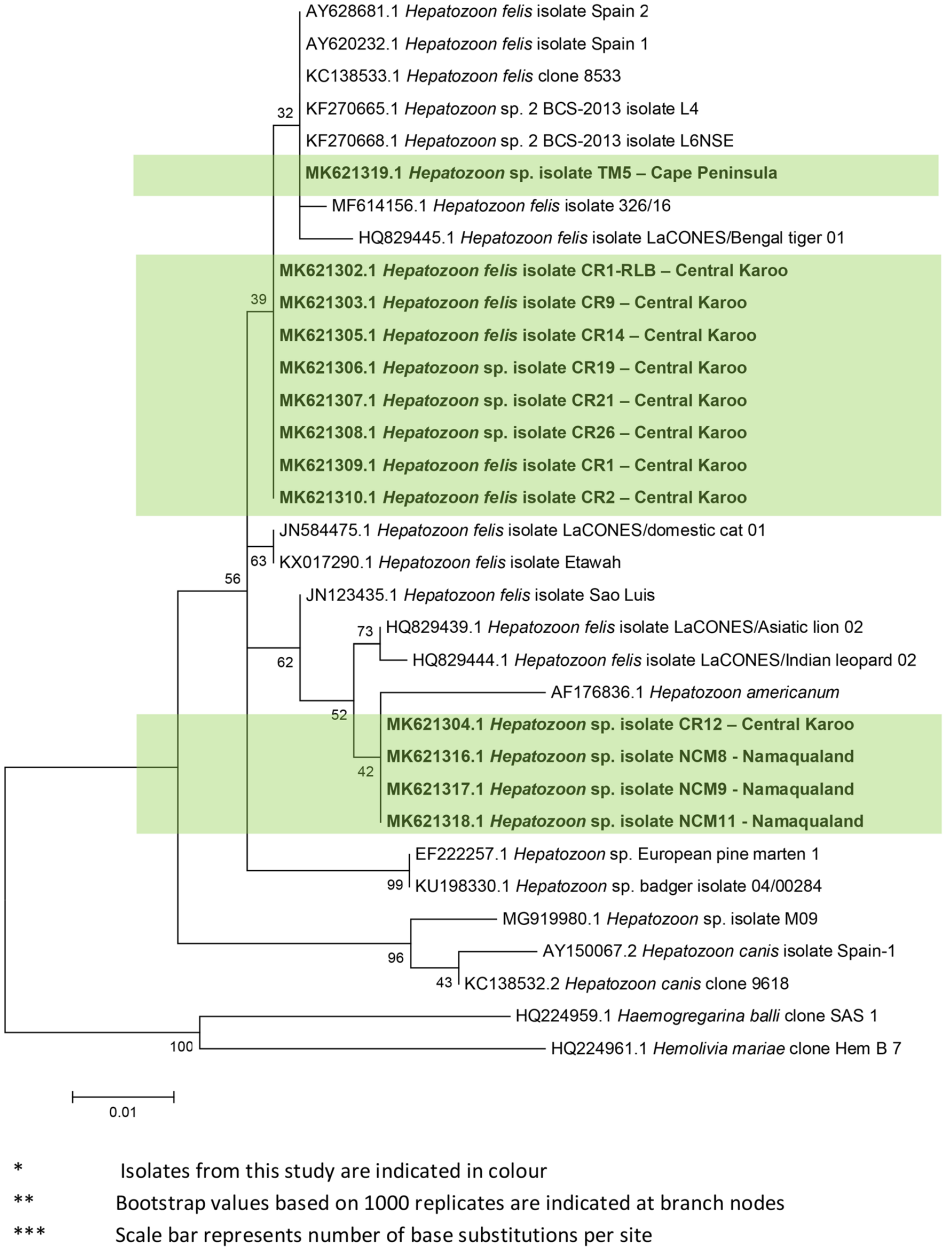
Maximum likelihood phylogeny (T92 + G substitution model) of partial *Hepatozoon* spp. *18S* rRNA gene sequences (531 bp)

Results from both BLASTn analysis and phylogenetic reconstruction of directly sequenced PCR products (Figs. 1, 2, 3) indicate that *Ehrlichia* spp. were not present in our study populations while *Anaplasma* spp. were only confirmed in the Cape Peninsula. According to BLASTn analysis, our isolated sequences had 99% identity to an *Anaplasma phagocytophilum*-like species isolated from domestic dogs in South Africa (GenBank: AY570538, AY570539 [79]), and from a Mongolian gazelle (*Procapra gutturosa*) in China (GenBank: KM186950). Despite a positive RLB result for the presence of *Ehrlichia/Anaplasma* in samples from the Central Karoo population, direct sequencing of samples did not support this result.

The BLASTn identification of a domestic dog *Anaplasma* sp. in Peninsula caracals is further supported by the phylogenetic placement of sequences within a well-supported clade comprising *Anaplasma* spp. sequences isolated in South African domestic dogs (Fig. 1). Results from both neighbour-joining and maximum likelihood tree construction methods recovered the same topologies for all analyses reported in this study, thus only maximum likelihood trees are presented here. Within the *Anaplasma* clade, the erythrocytic *Anaplasma* species (*A. marginale*, *A. centrale* and *A. ovis*) are distinct from the leucocytic *Anaplasma* species (*A. phagocytophilum*, *A. platys* and *A. bovis*), which is in agreement with the accepted *16S* rRNA phylogeny of the *Anaplasmataceae* [49, 74, 80].

#### Piroplasmid infections

All caracals showed hybridisation to the *Babesia* 1 genus-specific probe, while 96% (*n* = 55) also tested positive for the *Babesia* 2 probe. Both the *Babesia* 1 and *Babesia* 2 probes are specific to genus level, but differ in the regions they target. Fourteen caracals (25%) had *Babesia* species-specific binding, all of which were from the Cape Peninsula. Two of the sixteen (12.5%) Cape Peninsula caracals indicated no *Babesia* species-specific binding. According to the RLB, hybridization to multiple species-specific probes, including *B. felis*, *B. microti* and *B. leo*, occurred among Cape Peninsula caracals, with samples from 9/16 individuals (56%) hybridising to all three, 3/16 (19%) hybridising to *B. felis* and *B. microti* and one sample (6%) hybridising to *B. microti* and *B. leo*. One caracal showed hybridization only to *B. microti*, but not to either *B. felis* or *B. leo.*

Phylogenetic analysis of *Babesia* sequences isolated from our study populations supported the RLB results, placing *Babesia* isolates from caracals into two main clades: “*Babesia* (*sensu lato*)” contains isolates in the *Babesia felis*, *B. leo* and *B. microti/vulpes/rodhaini* groups, while “*Babesia* (*sensu stricto*)” contains isolates comprising *B. ovis*, *B. canis*, *B. gibsoni*, *B. odocoilei*, *B.* “*venatorum*” (following [81]). Sequences from the Cape Peninsula and the Central Karoo were placed in “*Babesia* (*sensu lato*)”, while sequences from Namaqualand and the Central Karoo were placed in “*Babesia* (*sensu stricto*)” (Fig. 2).

Infections with piroplasmid species were also confirmed by phylogenetic analysis. Sequence data from direct sequencing of PCR products confirmed infection by species of *Babesia* in all three study populations. Cape Peninsula caracals are infected with numerous *Babesia* species. *Babesia felis* was identified in two peninsula caracals, with sequence similarity of 98–99% with *Babesia* sp. identified in caracals from Kruger National Park, South Africa (GenBank: AF244914, AF244913) and from a domestic cat (*Felis catus*) in Port Elizabeth, South Africa (GenBank: AY452699). *Babesia leo* was identified in three of the peninsula caracal individuals, with 97–100% sequence similarity to *B. leo* isolated from a lion in Kruger National Park (GenBank: AF244911) and a domestic cat in Port Elizabeth, South Africa (GenBank: AY452708).

Direct sequencing of PCR products that targeted the genus *Babesia* also identified caracals infected with the closely-related pathogen, *Hepatozoon felis*. BLASTn analysis indicated 99–100% sequence similarity with *Hepatozoon* isolates from a range of felids including the domestic cat, African lion, Asian lion (*P. leo persica*) and Asian leopard (*P. pardus fusca*). A further *H. felis* sequence obtained from a Namaqualand caracal showed 99–100% sequence similarity to *H. felis* isolated directly from several tick species found on leopard cats (*Prionailurus bengalensis euptilura*) from Japan. Phylogenetic analysis of *Hepatozoon* isolates from caracals confirmed these results (Fig. 3); sequences from caracals were distributed across two different clades comprising both domestic cat isolates as well as those reported from a Bengal tiger (*P. tigris tigris*), Asian lion and Indian leopard. Piroplasms of *Cytauxzoon* species were included in “*Babesia* (*sensu stricto*)” [59] and would also be detected by the generic RLB piroplasm probes. None of the isolates selected for species identification were found to be *Cytauxzoon* species.

### Tick-borne pathogen prevalence in South African caracal populations

In the Central Karoo, three TBPs were confirmed in the caracal population (Table 2). *Hepatozoon felis* was observed at a prevalence of 92.6% (95% CI: 75.7–99.1%), *Babesia felis* at 7.4% (95% CI: 0.9–24.3%) and an unknown *Babesia* sp. was observed in a single caracal (3.7%; 95% CI: 0–19.0%). Mixed infections were identified in four individuals (14.8%; 95% CI: 4.2–33.7%). Namaqualand caracals showed similar prevalence patterns, with *H. felis* observed in 85.7% (95% CI: 57.2–98.2%) of individuals. However, the only *Babesia* species identified in individuals from this region appears to be a novel species, closely related to *B.* “*venatorum*”, and occurring at a prevalence rate of 14.3% (95% CI: 1.8–42.8%). No mixed infections occurred in Namaqualand caracals.

**Fig. 4:**
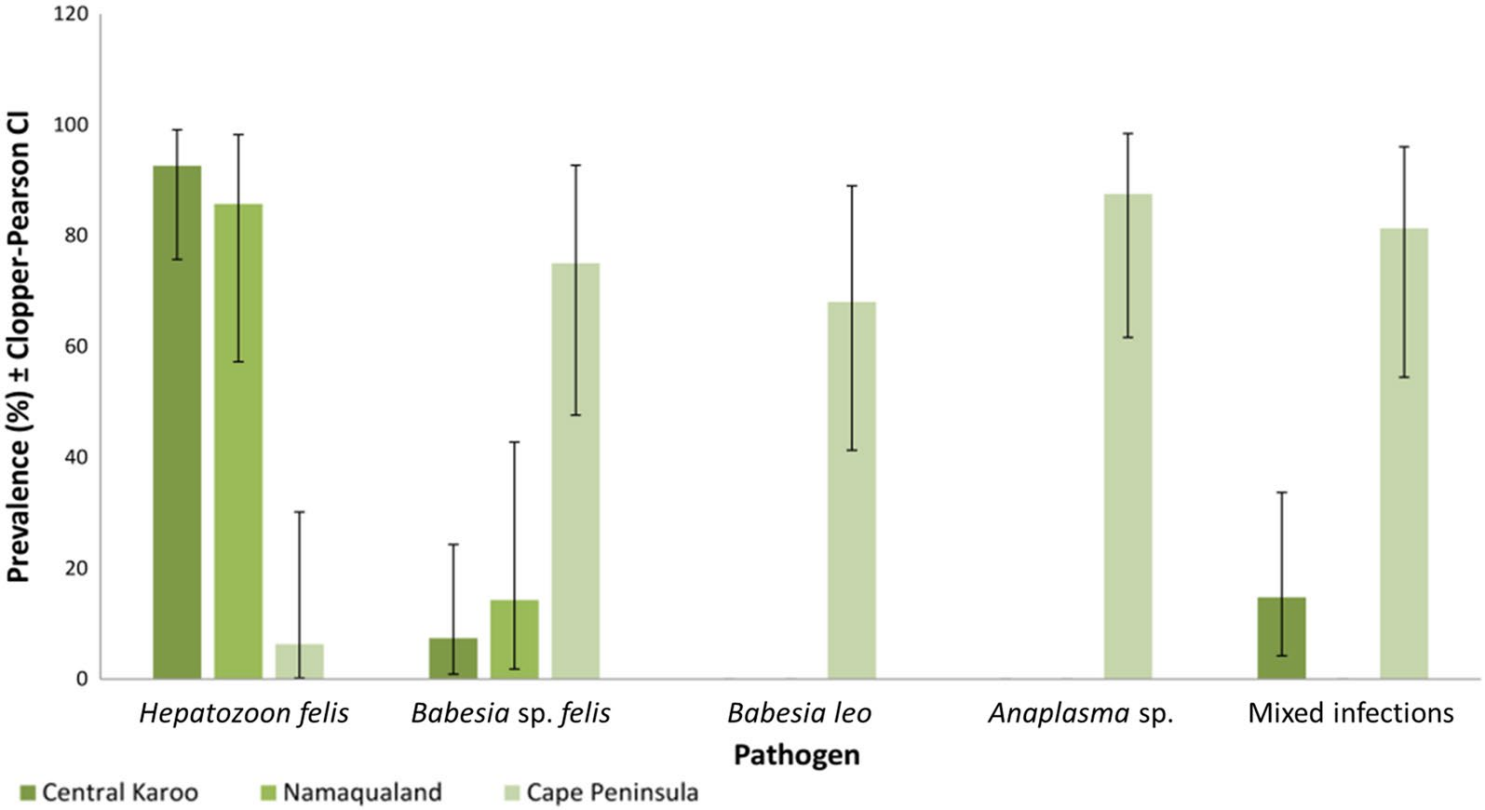
Tick-borne pathogen prevalence (%) in South African caracals based on PCR and direct sequencing

Pathogen prevalence in caracals from the Cape Peninsula contrasted with both the Central Karoo and Namaqualand (Fig. 4). Cape Peninsula caracals showed evidence of infection with *H. felis*, *B. felis*, *B. leo* and an *Anaplasma phagocytophilum*-like species. Rangeland caracal populations (Central Karoo and Namaqualand), supported similar prevalence of *H. felis* and *B. felis*, while the prevalence of *Hepatozoon felis* (6.3%; 95% CI: 0.2–30.2%) was lower for the Cape Peninsula caracals (*χ*^2^ = 74.864, *df* = 2, *P* < 0.001). Prevalence of *B. felis* (75%; 95% CI: 47.6–92.7%) was higher in the Cape Peninsula *versus* both the Central Karoo and Namaqualand (*χ*^2^ = 124.39, *df* = 2, *P* < 0.001). *Babesia leo* was only observed in the Cape Peninsula, where 68% (95% CI: 41.3–89%) of the sampled population was infected. The Cape Peninsula population is the only one in which an *Ehrlichia* or *Anaplasma* species was observed, with *A. phagocytophilum*-like species, most similar to *Anaplasma* isolates from South African dogs, occurring in 14 caracals (87.5%; 95% CI: 61.6–98.4%). The rate of mixed infections was also highest in the Cape Peninsula (*χ*^2^ = 116.98, *df* = 2, *P* < 0.001), occurring in 81.3% (95% CI: 54.4–96%) of the sampled population.

### Host-attached tick presence in South African caracal populations

Ticks were collected from seven caracals in the Central Karoo, 11 in Namaqualand and 13 caracals in the Cape Peninsula, yielding a total of 63 ticks across all sites. Exhaustive tick surveys were not executed. All sampled ticks were from the family Ixodidae, and included representatives from four genera (*Amblyomma*, *Haemaphysalis*, *Ixodes*, *Rhipicephalus*) and at least six species (*A. marmoreum*, *H. elliptica*, *H. zumpti*, *I. rubicundus*, *I. pilosus*, *R. gertrudae* and *R. capensis*). In most instances, differentiation among *H. elliptica* and *H. zumpti* could not be established due to morphological similarity, and hence both species are classified under the *H. elliptica/H. zumpti* group.

The larvae of *A. marmoreum* were the most prevalent ticks on Central Karoo caracals (57% of caracals with attached ticks), with *I. rubicundus* and *H. elliptica/H. zumpti* also recorded. Namaqualand caracals also carried *I. rubicundus* and *H. elliptica/H. zumpti*, as well as *R. gertrudae*. Cape Peninsula caracals appeared to host the greater abundance (S. Viljoen, personal observation) and diversity of ticks, with recorded species being *H. elliptica/H. zumpti*, *R. gertrudae* and two species only observed in the Cape Peninsula, namely, *R. capensis* and *I. pilosus.*

## Discussion

Molecular analysis of tick-borne pathogens in caracals from South Africa suggests an important role for land-use in determining the diversity and prevalence of tick-borne pathogens. Clear differences in the patterns of prevalence and diversity of TBPs characterised caracals from different land-use areas of South Africa, with the greatest overall prevalence and diversity of pathogen occurring in caracals on the Cape Peninsula. While climate, vegetation characteristics and vertebrate host density are all important for tick vectors [82, 83], this study draws attention to how TBPs in free-ranging caracals varies across human-modified land-use types. All individuals analysed in the study were infected with at least one TBP, with caracals occurring in the urban/agricultural/natural matrix of the Cape Peninsula having a greater prevalence of multiple TBP co-infections.

### High prevalence and diversity of tick-borne pathogen infections in peri-urban caracals

While rangeland caracals were not infected with either *Ehrlichia* or *Anaplasma* species, caracals living at the urban edge on the Cape Peninsula showed high rates of infection with an *Anaplasma* species most similar to that previously reported in South African (SA) domestic dogs [74]. This species, ‘*Anaplasma* sp. SA dog’, is similar to *A. phagocytophilum*, and has only since been reported once in black-backed jackals (*Canis mesomelas*) in the Gauteng Province of South Africa [79]. The latter finding suggests that there may be unexplored diversity of TBPs in the Cape Peninsula region, but also more broadly in South Africa.

*Anaplasma phagocytophilum* is a generalist pathogen known to occur in domestic animals and wildlife, including carnivores, wild ruminants, reptiles, rodents and birds (see [84] for an extensive list of hosts). Our observation of ‘*Anaplasma* sp. SA dog’ in peri-urban caracals indicates a possible epidemiological link where caracals and domestic dogs co-occur. Epidemiological relationships based on common vector-borne pathogens between sympatric domestic and wild carnivore species have previously been proposed in red foxes (*Vulpes vulpes*) and domestic dogs in Portugal [30], and between mountain lions (*Puma concolor*) and domestic cats in California, USA [29].

The prevalence of *Anaplasma* sp. in the Peninsula caracals is substantially higher than levels previously reported in studies of wild felids, including lions, South African wildcats (*Felis silvestris cafra*), cheetahs and servals (*Leptailurus serval*) [38]. Our results revealed 88% prevalence in Peninsula caracals while a range of wild felid species from Zimbabwe showed an average prevalence rate of only 8% [38]. Felids outside of the African continent (*Puma concolor* and captive *Panthera leo*) have been reported with up to 10% infection rates of *A. phagocytophilum* [29, 85]. The small pool of survey data to which our data can be compared limits our ability to infer whether the high prevalence in the Peninsula caracals is unusual, and thereby of concern to wildlife managers. Our findings do, however, provide the first record of *Anaplasma* infection in caracals, making it one of very few records from wild felids in Africa (see [38]).

*Ehrlichia* sp. could not be confirmed in any of the samples that were positive for *Ehrlichia/Anaplasma* based on the RLB. This finding was not unexpected, as a number of studies that use molecular techniques to detect blood parasites in carnivores report similar findings [86, 87]. It is possible that the low prevalence of *Ehrlichia* parasites and/or the small sample size of most studies limit the detection of *Ehrlichia* presence in the host population.

#### Protozoon diversity across caracal populations

*Babesia* infections have been noted in both domestic and wild animals in many parts of the world [22, 87–89]. A number of these *Babesia* species have demonstrated zoonotic potential [90–92], while others are important disease-causing agents in domestic dogs, e.g. *B. canis* in Europe and *B. gibsoni* worldwide [93].

Our study identified a number of *Babesia* species known to infect both wild and domestic cats. In addition to ‘*Anaplasma* sp. SA dog’, Cape Peninsula caracals were also host to *B. felis* and *B. leo*, which have both previously been reported occurring in caracal from rural areas in KwaZulu-Natal, South Africa [15]. Phylogenetic analysis of the *Babesia* species circulating in Peninsula caracals suggests there is a large degree of *B. felis* diversity represented in the Cape Peninsula’s caracal population, possibly indicating multiple infections.

Caracals from the Central Karoo were also infected with *B. felis* together with an unknown *Babesia*, most similar to isolates of *B.* “*venatorum*”, which has yet to be comprehensively described. *Babesia* “*venatorum*” is considered an emerging zoonosis in Europe and Asia [40, 94], but has yet to be reported in Africa.

Direct sequencing of *Babesia*-positive individuals from all three populations revealed infection of individuals with the apicomplexan blood parasite *Hepatozoon felis.* To the best of our knowledge, this is the first report of *H. felis* in caracals. *Hepatozoon felis* was detected in all three caracal populations, and at least two strains of *H. felis* were identified in the individuals analysed in this study. Unlike their semi-arid rangeland counterparts, Peninsula caracals have a low prevalence of *H. felis*.

Central Karoo caracals do not support a wide diversity of TBPs, and the vast majority of the high overall prevalence of TBPs in this population is attributed to widespread infection with *H. felis*. The caracal population in Namaqualand revealed a similar TBP profile to the Central Karoo population. In these semi-arid landscapes, strains of *Hepatozoon felis* were the dominant TBP circulating in caracals. Based on phylogenetic analysis, one of these strains is very similar to *H. felis* isolated from domestic and wild felids across the globe, e.g. lions in Zambia, tigers (*Panthera tigris*) in India and domestic cats from Spain and Israel. This common strain was only identified in individuals from the Central Karoo. The second *Hepatozoon* clade is sister to the ‘canis-felis’ clade, and occurred in both the Central Karoo and Namaqualand caracals. Isolates in this second clade appear to be distinctive from those previously reported for *H. felis*.

Samples from two caracals hybridised to the *Theileria* genus-probe, with only one indicating species-specific binding to the *T. annulata* probe. Evidence for *Theileria* infection could not be confirmed by sequencing. The known distribution of *T. annulata* does not extend into the southern Africa [95], and thus this finding is most likely a cross-reaction. The detection of *Hepatozoon* species when using PCR primers that are designed for *Babesia* species is not uncommon, and has previously been noted by other authors (e.g. [96] in bank voles (*Myodes glareolus*) in Germany).

There was not enough variation in pathogen prevalence to examine drivers of infection using a Generalized Linear Model (GLM) framework (e.g. [97]). The use of logistic regression modelling would be useful in attempting to answer questions relating to drivers of pathogen prevalence, which could greatly improve our understanding of epidemiology and tick-borne disease dynamics. However, large sample sizes and a variety of predictor variables measured across sites are required.

### Tick diversity on caracals in human-modified landscapes

In sub-Saharan Africa, where tick-borne diseases are among the most important threats to livestock and domestic animals, very few tick species have been established as disease vectors [98]. Other than causing blood loss and/or biting stress associated with high tick burdens, *Ixodes pilosus*, *Amblyomma marmoreum* and *Rhipicephalus capensis* are not known to transmit any notable pathogens or be responsible for any severe toxicosis. However, *I. pilosus* is thought to be common on both domestic cats and caracals [99–101]. *Rhipicephalus gertrudae* has been reported to harbour the rickettsial pathogens, *Anaplasma centrale* and *A. marginale* [102], both of which are economically important for the livestock industry.

The only tick species observed in all three sites, and also which we observed as the most common (data not shown), are those in the *H. elliptica/H. zumpti* group. *Haemaphysalis elliptica* is considered the only tick of veterinary importance which infects both domestic and wild felids [101] and is also suspected of being the vector for *B. felis* and *B. leo*, although this has yet to be demonstrated conclusively. The high prevalence of *B. felis* and *B. leo* in Cape Peninsula caracals provides some support for this hypothesis, but should not be interpreted as evidence of a vector role. *Haemaphysalis elliptica* is one of the most common ticks on South African domestic dogs [101, 103], as well as being abundant in domestic and wild felids in South Africa [100, 104]. Both *H. elliptica* and the morphologically similar *H. zumpti* have previously been recorded in caracals [101]. Interestingly, *H. elliptica* is also considered the vector of *Babesia rossi*, the causative agent of canine babesiosis [105]. Given that domestic dogs are present in all study sites, particularly in the urban matrix of the Cape Peninsula, the ubiquity of this tick species likely has important consequences for the spread of TBPs in the sites examined.

The similarity in tick presence between the Central Karoo and Namaqualand tick is likely as a result of the similar climate, vegetation structure and host communities that exist in both sites [83]. Caracals from the Cape Peninsula differ from the rangeland caracals in that they appear to host a greater diversity of ticks. However, given that tick sampling was not exhaustive this finding is anecdotal.

## Conclusions

This study represents the first molecular survey of TBPs in free-ranging caracals in South Africa. The findings presented here contribute to the growing body of work on tick-borne pathogens infecting wild carnivores, and specifically to our knowledge of carnivores living in human-modified landscapes. These data represent an exploratory investigation into the pathogen diversity present in South African caracals. Baseline data on pathogen diversity and prevalence in wildlife is generally lacking worldwide, even in systems such as the human-wildlife-livestock interface, where this knowledge is of economic value. Our results demonstrate potential epidemiological links that wild carnivores persisting in human-modified landscapes may have with sympatric domestic species. The extent of the influence that land transformation has on the health of wild carnivores in sub-Saharan Africa remains largely unknown, but extensive screening of important pathogens is the first step in improving regional understanding of wildlife disease ecology within the One Health framework.

## Data Availability

Data analysed for this publication are available from the corresponding author on request. All original sequences have been accessioned in the GenBank database: MK271634-MK271635 (*Anaplasma* spp.); MK616186-MK616205 (*Babesia* spp.) and MK621302-MK621310 and MK621316-MK621319 (*Hepatozoon* spp.).

## Abbreviations

TBP: tick-borne pathogen(s)
rRNA: ribosomal ribonucleic acid
MAP: mean annual precipitation
EDTA: ethylenediaminetetraacetic acid
RLB: reverse line blot
PCR: polymerase chain reaction
DNA: deoxyribonucleic acid
SSPE: saline sodium phosphate EDTA
SDS: sodium dodecyl sulfate
BLASTn: Basic Local Alignment Search Tool for nucleotides
AICc: Akaike’s information criterion with small sample correction
CI: confidence interval
K2: Kimura 2-parameter model
G: Gamma distribution
T92: Tamura 1992 model
I: invariable sites
df: degrees of freedom
SA: South African
GLM: Generalised linear model
